# COVID-19 Like Findings in a Fatal Case of Idiopathic Desquamative Interstitial Pneumonia Associated With IgA Glomerulonephritis in a 13-Month-Old Child

**DOI:** 10.3389/fped.2020.586666

**Published:** 2020-11-11

**Authors:** Simona Gurzu, Catalin Bogdan Satala, Lorena Elena Melit, Adrian Streinu-Cercel, Dan Otelea, Brandusa Capalna, Claudiu Ioan Puiac, Janos Szederjesi, Ioan Jung

**Affiliations:** ^1^Department of Pathology, George Emil Palade University of Medicine, Pharmacy, Sciences and Technology, Targu-Mures, Romania; ^2^Department of Pathology, Clinical County Emergency Hospital, Targu-Mures, Romania; ^3^Department of Microscopy, Research Center of the University of Medicine, Pharmacy, Sciences and Technology, Targu-Mures, Romania; ^4^Department of Pediatrics, Clinical County Emergency Hospital, Targu-Mures, Romania; ^5^Department of Pediatrics, George Emil Palade University of Medicine, Pharmacy, Sciences and Technology, Targu-Mures, Romania; ^6^National Institute for Infectious Diseases “Matei Bals”, Bucharest, Romania; ^7^Department of Intensive Care, George Emil Palade University of Medicine, Pharmacy, Sciences and Technology, Targu-Mures, Romania

**Keywords:** infant, interstitial pneumonia, autopsy, SARS, COVID, glomerulopathy, IgA, Berger

## Abstract

In the COVID-19 era, patients with severe acute respiratory syndrome (SARS) are suspected to be associated with SARS-CoV-2 infection. The aim of this paper is to present a case with COVID-like pneumonia, with fatal evolution. The clinical aspects were correlated with the autopsy findings and discussed on the background of the most recent data from the medical literature. A 13-month-old girl was admitted to the emergency room with acute severe shortness of breath and pulmonary bilateral ground-glass opacities and an almost complete opacified left lung. The patient suddenly deteriorated, and death was confirmed 3 h after admission. At autopsy, severe desquamative interstitial pneumonia was diagnosed and was associated with an unusual IgA glomerulonephritis. No SARS-CoV-2 infection was detected in the lung parenchyma by RT- PCR. This is a very unusual case of rapid deterioration of an infant with idiopathic desquamative interstitial pneumonia (IDP) and multiorgan involvement. Based on immunohistochemical stains, we hypothesize that, in IDP, the hyaline membranes arise from necrotizing desquamated pneumocytes. In the COVID-19 era, such cases are extremely difficult to diagnose; they can mimic SARS-CoV-2-induced lung injuries. This pattern of hyaline membrane formation might explain the poor response to oxygen therapy. The present case highlights the importance of autopsy in such challenging cases.

## Introduction

Desquamative interstitial pneumonia (DIP) is a rare disorder: only 362 cases have been reported in adults, mostly among smokers (1). In children, its etiology and pathomechanism were far from reaching consensus in the 41 English-language articles found in the literature until 2020 (1–3). To the best of our knowledge, only three of the reported DIP cases in children presented with associated kidney failure (4), and not one featured rapid and fatal deterioration in patients below 2 years old.

In children, DIP is thought to be caused by an inborn defect in surfactant metabolism, which is associated with progressively developing respiratory symptoms and a negative impact on growth (2, 3). It is believed to have an immune-mediated pathogenesis (4) and to predispose to pulmonary fibrosis (3).

In this paper, we present an unusual case of DIP diagnosed in a 13-month-old girl, who died a few hours after hospital admission. There are a few aspects that make this case unique in the literature. First, it started in an apparently healthy baby with a rapid deterioration of health status. Second, the association with a proliferative glomerulonephritis at such an early age has not yet been described in the literature. Then, because the case was diagnosed during the COVID-19 (coronavirus disease 2019) pandemic, the histological picture of the clinical and the morphological signs corresponded to severe acute respiratory syndrome (SARS), a SARS-CoV-2 infection was suspected; however, this was not confirmed by repeated RT-PCR tests. The case highlights some of the challenges pediatrics departments may face soon as well as the importance of autopsy in difficult cases that might mimic a SARS-CoV-2 infection. Along with the case presentation, a review of recent literature regarding the possible COVID-induced clinical and morphological features is presented together with a paradigm about hyaline membrane formation in cases of DIP. This latter fact might explain the poor response to oxygen therapy in COVID-induced and COVID-like cases.

To the best of our knowledge, the present case is the fourth case of DIP with renal involvement described in a child in the English literature, and the first with fatal evolution, possibly as a result of an associated SARS-CoV-like infection. Signed informed consent of the mother was obtained for the publication of this case.

## Case Report

During a nightshift at our emergency room, a 13-month-old girl was referred because of cough, chills, and serious shortness of breath with fever (38°C). According to the medical history obtained from mother, no perinatal or post-birth growth or development abnormalities were reported. The deterioration started a few hours before the present admission, and possible contact with a SARS-CoV-2-infected person was denied.

A physical examination revealed pale skin and mucosae and a hypotonic baby with hepato-splenomegaly, tachypnea, tachycardia, and oxygen saturation of 80%. Severe dyspnea demanded the emergency use of an oxygen face mask. Intubation was performed after only a few minutes. No abnormalities were identified on ECG and abdominal ultrasound.

Blood analysis indicated dehydration and severe anemia - with a very low serum level of hemoglobin (1.5 g/Dl) and low hematocrit (6.7%). Mild uremia (39.8 mg/Dl) and slightly decreased serum creatinine (0.36 mg/Dl) were considered indicators of dehydration. A mildly increased serum in C-reactive protein level (14.6 mg/L) was also noted (Table 1). The urine analysis showed leukocyturia but not bacteriuria.

**Table 1:** Blood parameters are indicators of severe anemia, undernourishment and dehydration.

**Parameter**	**Patient's value**	**Normal range**
Leukocytes (×10^3^/μL)	8.21	4–12
Hemoglobin (g/dL)	***1.5***	10–14.2
Erythrocytes (×10^6^/μL)	***1.27***	3.5–4.5
Hematocrit (%)	***6.7***	36–47
Medium cellular volume – MCV (fL)	***52.8***	80–98
Neutrophils (×10^3^/μL)	3.06	1.5–8.5
Lymphocytes (×10^3^/μL)	4.42	3–10.5
C-reactive protein - CRP (mg/L)	***14.6***	<5
Urea (mg/dL)	***39.80***	10.51–35.55
Creatinine (mg/dL)	***0.36***	0.57–1.11
Aspartate aminotransferase - AST (U/L)	36	5–34
Alanine aminotransferase - ALT (U/L)	25	0–55

*Bold and italic values represent the abnormal (modified) ones, which were modified - below or up to the normal range*.

**Figure 1:**
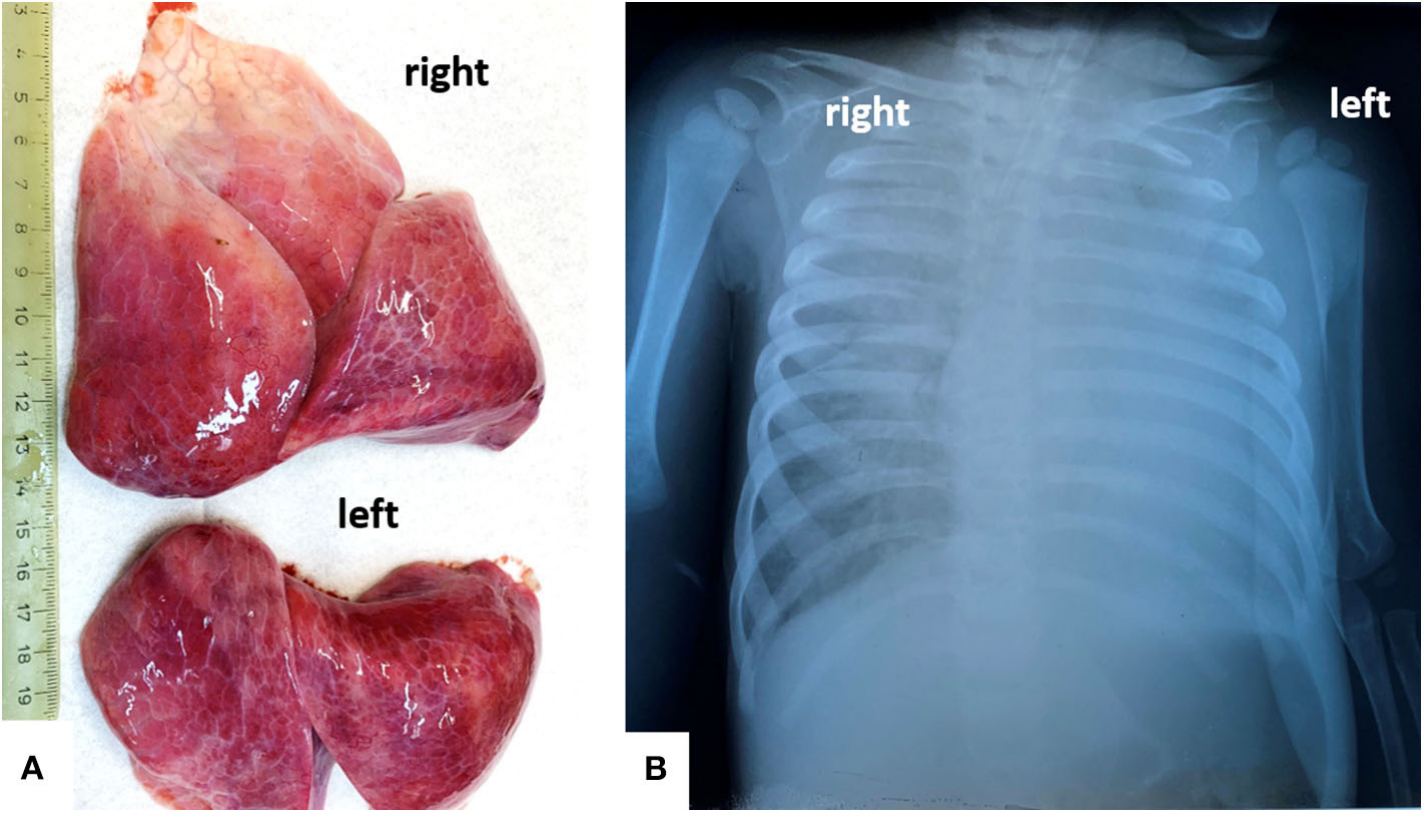
In a 13-months old girls, bilateral severe pulmonary dystelectasis, revealed by post-mortem examination **(A)**, correspond to the Rx-image, which shows ground-glass opacities, in the right lung, and diffuse opacity of the left lung **(B)**.

A chest X-ray revealed almost complete opacity in the left lung and patchy infiltrates in the right lung (Figure 1). Despite intravenous fluid boluses, followed by maintenance intravenous fluid therapy and supportive care—including oxygen therapy—death was confirmed 3 h after admission to the hospital.

Due to the sudden respiratory compromise associated with chest X-ray findings, a SARS-CoV-2 infection was suspected. Based on Romanian law, autopsy was indicated because no nasopharyngeal swab test was done.

Signed informed consent to perform the autopsy and publish the scientific results was obtained from the mother. The mother declared that she did not visit the hospital for a pre- and post-natal follow-up and did not adhere to the programmed vaccination schedule. The contact tracing did not identify possible contact with SARS-CoV-2 infection, but a complete medical history was difficult to obtain from the mother.

### Autopsy Findings

To reduce the infective risk, a trained technician together with the Head of the Department of Pathology (GS) performed the post-mortem examination in the autopsy room. They used complete personal protective equipment.

The external examination revealed a baby with extremely pale skin who seemed to be normally developed (height: 88 cm; weight: 10 kg) but who showed generalized edema without pitting. During *in-situ* examination of the organs, a normal thymus, bilateral pleural (50/50 ml) and pericardial effusions (30 ml), hepatomegaly (536 g), splenomegaly (64 g), and intestinal pneumatosis were described.

**Figure 2:**
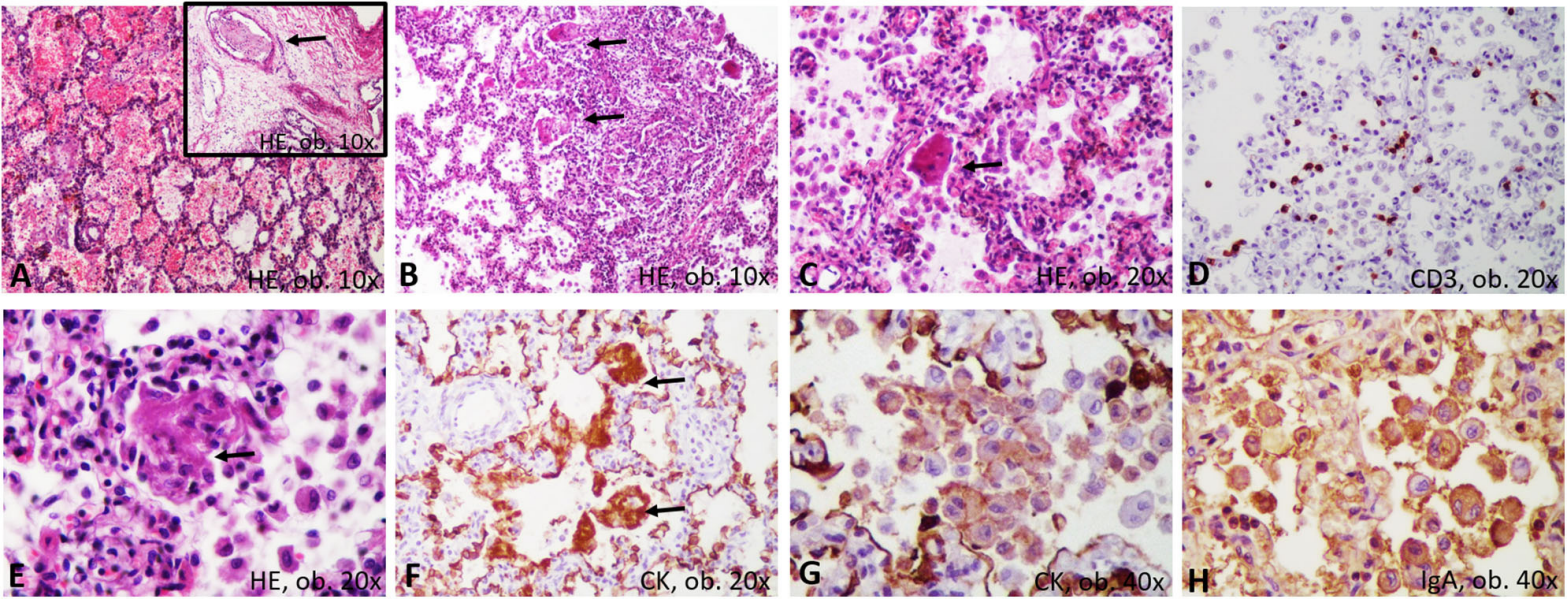
Microscopic and immunohistochemical features of desquamative interstitial pneumonia: **(A)** hemorrhages and thrombi (arrow); **(B)** bronchiolar damage; **(C)** desquamated pneumocytes, with syncytial-like bodies; **(D)** scanty T-cells; **(E)** necrosis of the desquamated cells, with genesis of membranes; **(F)** Encasement of alveolar septa by cytokeratin-positive membranes; **(G)** Positivity of the desquamated cells for cytokeratin; **(H)** IgA positivity in the desquamated cells.

Both lungs were enlarged, firm, and dystelectatic (136 g left; 169 g right) and without friable areas; the cut section was without gross features suggestive of bronchopneumonia (Figure 1). Under a microscope, the pulmonary sample examination showed bilateral diffuse alveolar damage (DAD) with severe desquamation of type II alveolocytes (pneumocytes) and the presence of intra-alveolar macrophages (Figure 2). A synchronous CD68/IgA/Cytokeratin AE1/AE3 (CK) positivity among the desquamated cells was proven without immunohistochemical (IHC) expression for the markers of Langerhans histiocytosis, CD1a and polyclonal S100. Scant interstitial mononuclear infiltration with a predominance of lymphocytes was also observed. The bronchial epithelium was focally damaged and expressed IgA positivity. Desquamative bronchiolitis was associated. Small foci of extravasated erythrocytes were also described along with vascular thrombi (Figure 2).

**Figure 3:**
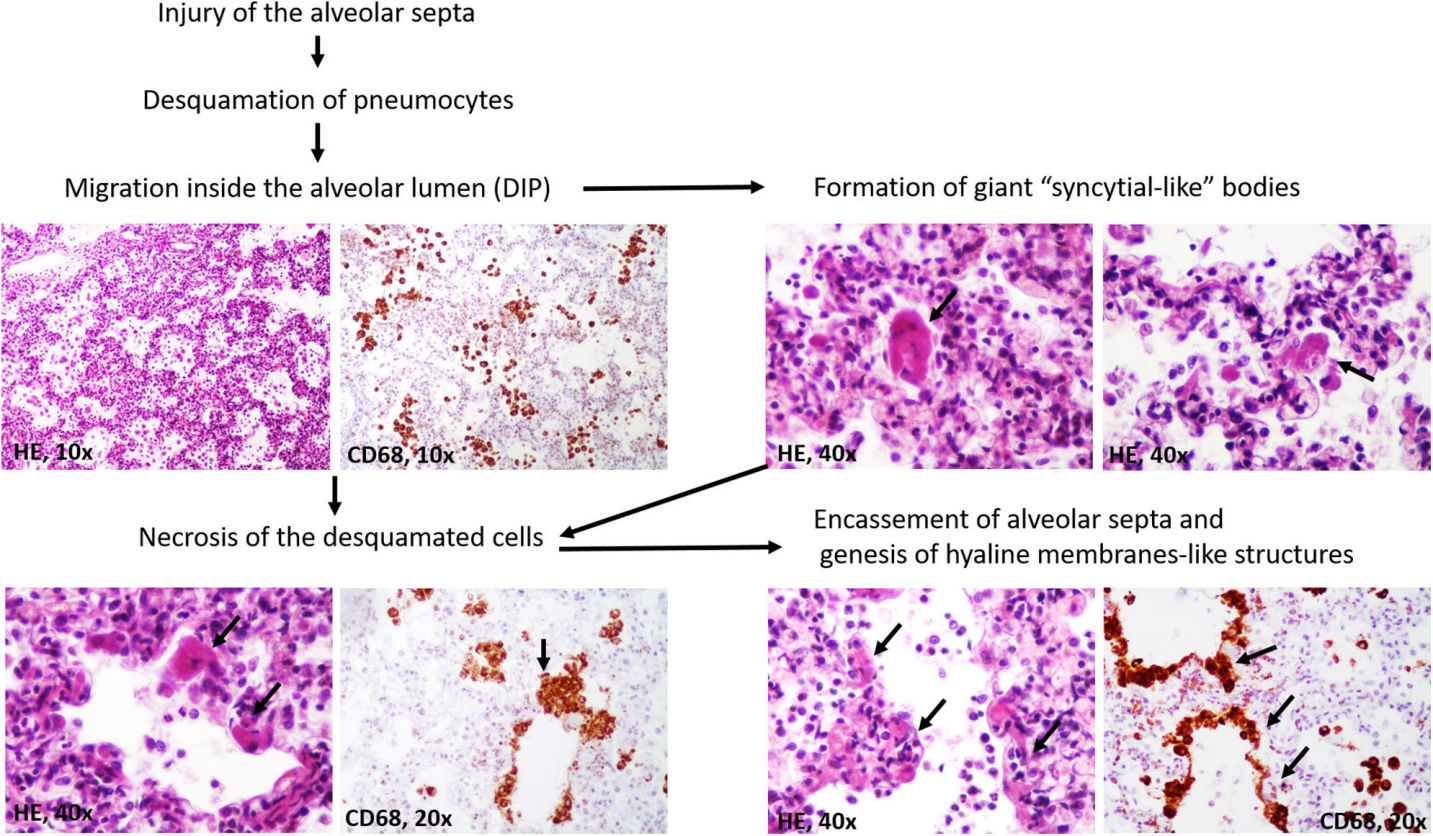
Stepwise genesis of hyaline-membrane-like structures, in desquamative interstitial pneumonia (DIP).

A particular aspect regarded the possible stepwise formation of hyaline membranes from CD68/CK-positive desquamated pneumocytes (Figure 3). They were predominantly seen inside the alveoli, together with macrophages. Most of the desquamated pneumocytes presented as round or elongated uninucleate cells. Some were enlarged, showing eosinophilic cytoplasm, and the nuclei were partially pushed to the periphery. They mimicked “syncytial cells.” On the other hand, within the alveoli septa, we observed some CD68/CK-positive, necrotic, enlarged pneumocytes, the fusion of which led to the genesis of CD68/CK-positive hyaline membrane-like structures with alveolar septa encasement. Inside the alveoli, few neutrophils were in the neighboring membranes (Figures 2, 3).

Two fresh tissue samples from the pulmonary dystelectatic areas were transported for real-time PCR assay to Cantacuzino National Research and Development Institute for Microbiology and Immunology (Romania). Although the histological features might correspond to SARS, the PCR assay did not confirm SARS-CoV-2 involvement in the above-described lung injuries. Molecular examinations performed at the National Institute for Infectious Diseases Matei Bals (Bucharest, Romania) on formalin-fixed, paraffin-embedded lung tissue also failed to detect by PCR SARS-CoV-2 as well as influenza A and B and respiratory syncytial virus (RSV).

**Figure 4:**
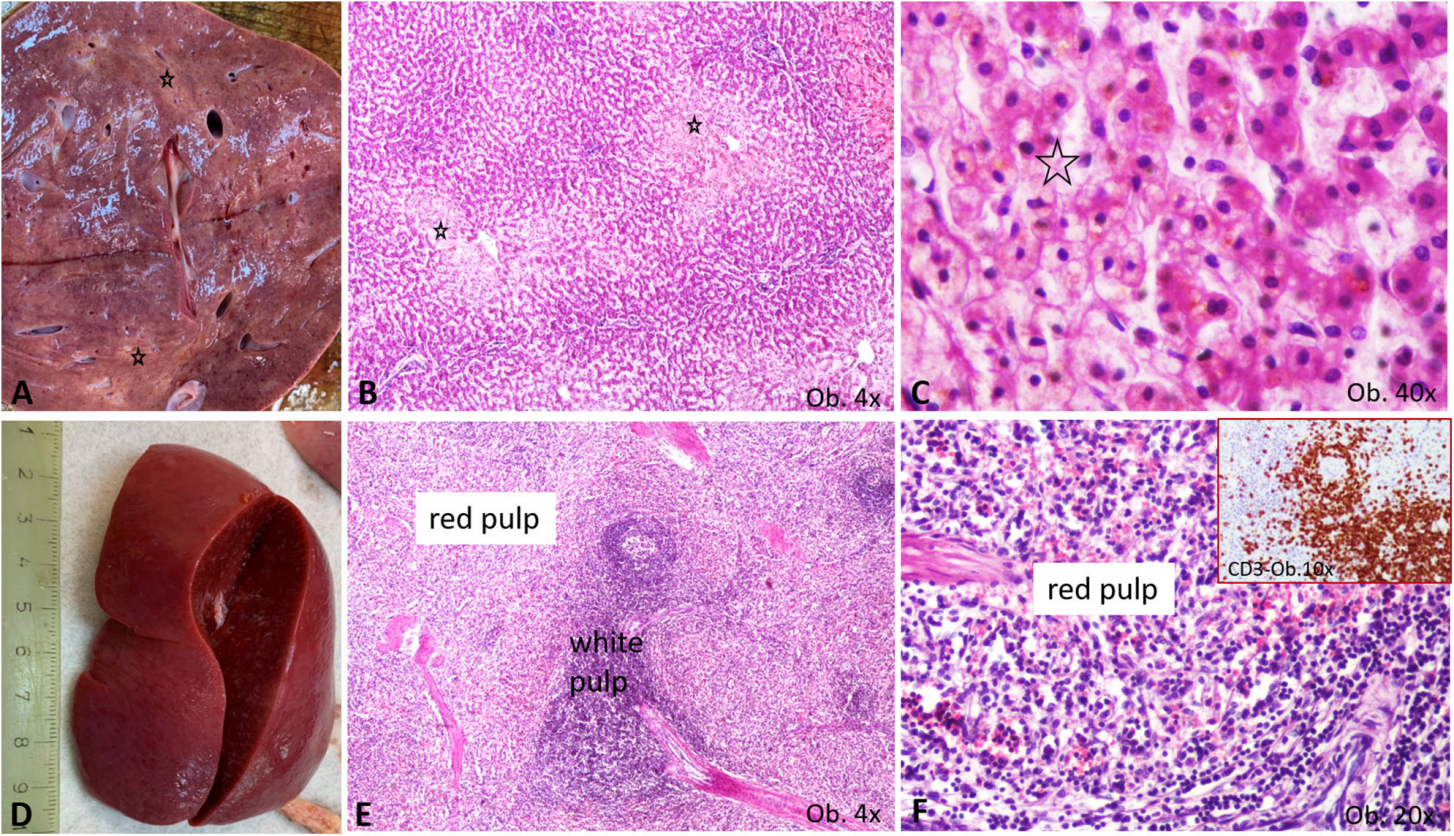
Gross and microscopic features of liver and spleen. **(A–C)** Liver centrilobular fatty change, with pale macro- **(A)** and microscopic area **(B)**, which show a multivacuolar aspect, at high power view **(C)**, being marked by *; **(D–F)** Splenomegaly specimen **(D)** is characterized by enlargement of the white pulp **(E)**, with T cell-rich aspect of the red pulp **(F)**, proved by the CD3 positivity (**F** corner).

Regarding the other organs, the severe brain edema (weight 960 g) was associated with meningeal hyperemia and the dilatation of the lateral ventricles without signs of meningitis or encephalitis. In the enlarged liver, centrilobular vesicular degeneration was seen under a microscope. In the enlarged spleen, mild hyperplasia of the white pulp together with CD3-positive T cell infiltration of the red pulp was described (Figure 4). T-cell hyperplasia of the paracortical area was also observed in the pericecal mesenteric lymphadenopathies. No cardiac or other malformations were identified, and myocarditis was not seen.

**Figure 5:**
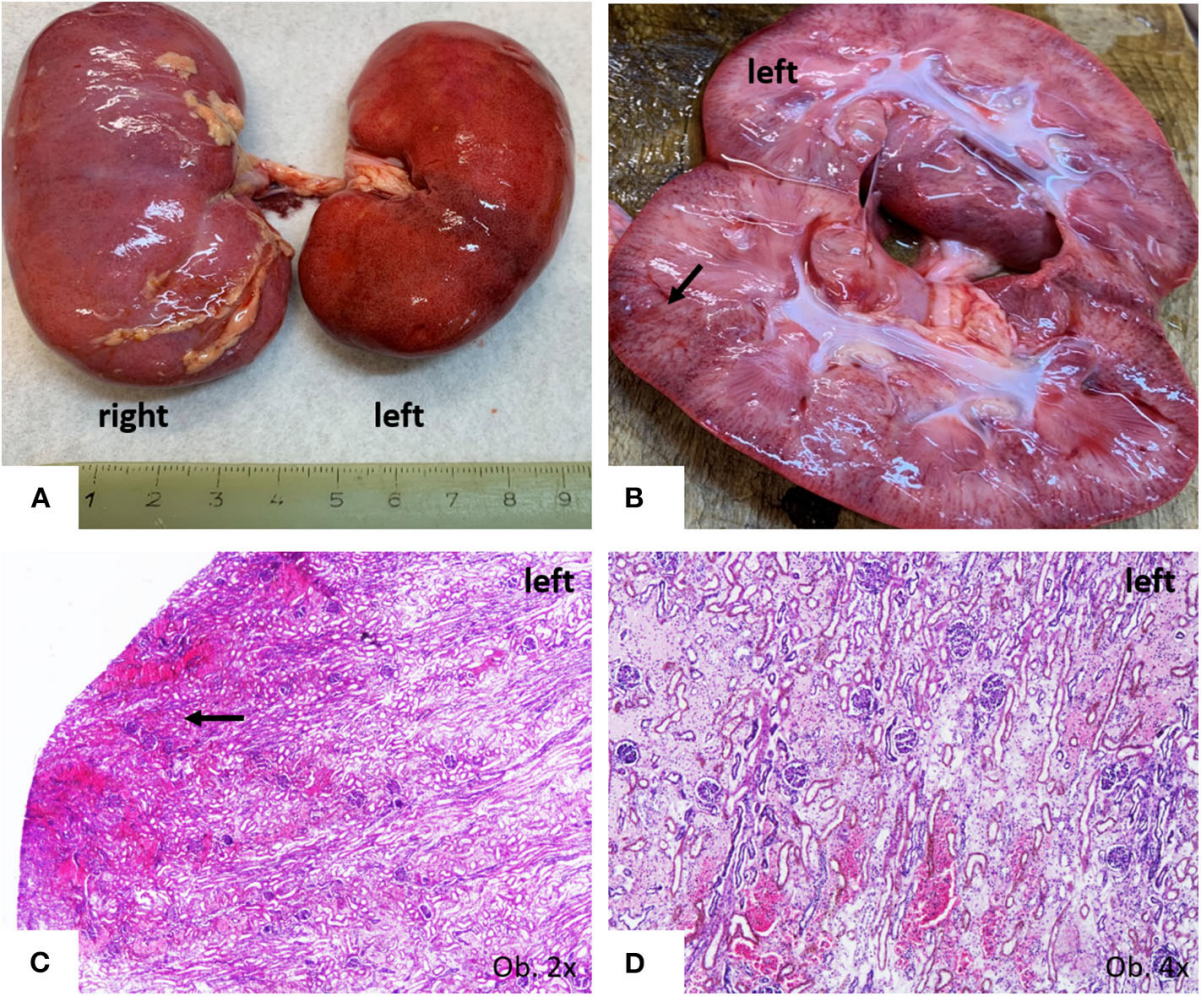
Gross findings of kidneys. **(A)** Bilateral enlargement, with hyperaemic surface of the left kidney; **(B,C)** Passive hyperemia of the cortex; **(C,D)** Parenchymatous congestion, with interstitial edema.

**Figure 6:**
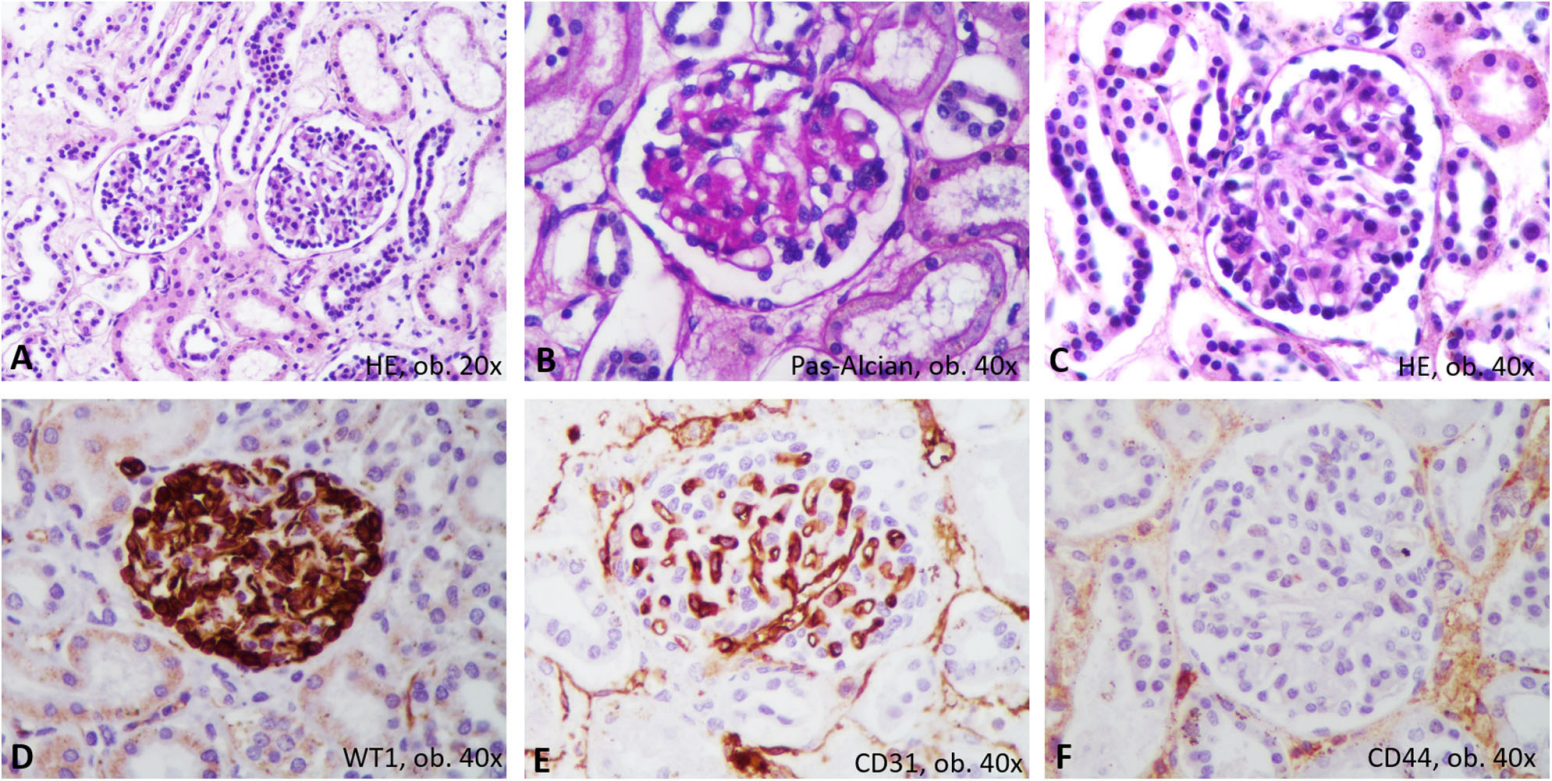
Proliferative glomerulonephritis. In Hematoxylin-Eosin, microscopic features consist on mesangium enlargement and proliferation of podocytes **(A–C)**. Immunohistochemically, the proliferated podocytes are marked by WT1 **(D)** and surround the CD31-positive capillaries **(E)**, without positivity for CD44 **(F)**.

The unusual bilateral enlargement of the kidneys (both weighed 146 g) called for a further detailed examination. Macroscopically, the left kidney was clearly hyperemic with a predominance of cortex hyperemia on the cut section (Figure 5). Under a microscope, most of the glomeruli showed enlargement of the mesangium with storage of IgA-positive cells and the proliferation of WT1-positive podocytes, without modifications to the cells of Bowman's capsule; this fact was confirmed by negative staining for CD44 (Figure 6). Interstitial nephritis with mononuclear cells was also described. The macroscopically hyperemic cortex showed dilatation of the veins under microscope (Figure 5), which might be the effect of blood flow impairment.

Based on the morphological, histological and IHC aspects, the postmortem diagnosis was “DIP associated with IgA proliferative glomerulonephritis, possibly occurring in the context of a SARS-CoV-like infection.” Death was the result of multiorgan failure syndrome (MOFS), which was probably initiated by severe lung injury.

## Discussion

The first two cases of DIP with associated chronic kidney failure in children were published in 1992 (4). In one, DIP was diagnosed at 10 months based on a lung biopsy, with proteinuria occurring at age 5, and focal segmental glomerulosclerosis occurring at 16, which required hemodialysis (4). The third case of idiopathic DIP with extrapulmonary manifestations was published in 2014 and presented in a 30-month-old girl (2). We did not identify other similar cases occurring in infants.

In pre-COVID times, DIP with IgA glomerulonephritis with rapid deterioration in an infant with severe anemia, dehydration, and poor feeding, such in the present case, would be considered a rare pediatric presentation (2). However, in the COVID-19 pandemic, rapid pulmonary decompensation—despite the absence of an epidemiological background, which was difficult to obtain based on family medical history—forced us to include the case as a “suspect case.”

The COVID-19 pandemic has affected millions of people worldwide, but cases in children are rare. Moreover, in the few cases reported in the literature, 90% of COVID-positive children had a milder form of the disease when compared to adults and deaths were extremely rare (5, 6). The clinical spectrum resembles that of influenza involving fever, cough, sneezing, sore throat, fatigue, and myalgia (7–9). Atypical presentations with gastrointestinal manifestations, such as vomiting or diarrhea, and malnutrition, were also described in children (9). Low oxygen saturation (<92%) was pointed out in fewer than 5% of cases as compared to tachypnea and tachycardia, which were present in substantial proportions (28.7 and 42.1%, respectively) at the time of hospital admission (9). Even though COVID-19 was not confirmed in the above case, our patient presented with both cough and fever but also with severe shortness of breath and low oxygen saturation, suggesting the possibility of a poor outcome.

Another rare disorder which should be considered for differential diagnosis of such cases is known as the Pediatric Inflammatory Multisystem Syndrome: Temporarily Associated with SARS-CoV-2 (PIMS-TS) (10–12). Rare cases were registered in Europe and North America expressing features like other pediatric inflammatory disorders, such as Kawasaki disease, bacterial sepsis, toxic shock syndrome, or macrophage activation syndrome. PIMS-TS is known to be associated with myocardial dysfunction, lesions of the coronary arteries, gastrointestinal and systemic involvement (10). According to the Royal College of Paediatrics and Child Health, PIMS-TS should be suspected in any child presenting with persistent fever, signs of inflammation, among which neutrophilia, increased C-reactive protein and lymphopenia, and of single or multi-organ dysfunction, such as shock, cardiac respiratory, renal, gastrointestinal or neurological conditions (11, 12). Our patient presented with fever, but the mother could not precisely time the onset of the fever, and therefore we were not able to delineate its persistence. Despite the presence of a clinical respiratory disorder and renal impairment proved by autopsy, both neutrophils and lymphocytes count were in normal ranges and the blood and urine culture were negative, as was the PCR testing for SARS-Cov-2, which might be negative or positive in PIMS-TS. In terms of imaging and ECG, PIMS-TS is associated with myocarditis, valvulitis, pericardial effusion, dilatation of the coronary arteries, patchy symmetrical infiltrates and pleural effusion on chest X-ray, and lymphadenopathies, ascites, hepatosplenomegaly or signs of colitis and ileitis on abdominal ultrasound. In our case, both ECG and abdominal ultrasound showed no pathological findings, which were confirmed at autopsy. Moreover, the chest X-ray findings were not suggestive since the patchy infiltrates were asymmetrical, the right lung being completely opacified, while patchy infiltrates were identified in the left lung. Based on all these facts, we consider that our patient's symptoms are most-likely not related to PIMS-TS.

In terms of laboratory parameters, children with COVID-19 seem to have relatively lower rates of lymphopenia and increased inflammatory biomarkers in comparison to adults (5). Therefore, a review that included 66 children from 12 studies revealed normal leukocyte counts in 69.2% of the cases, neutropenia in 6%, neutrophilia in 4.6%, and lymphopenia in 3%. However, the C-reactive protein was elevated only in 13.6% of these children (13). A mildly higher value of the C-reactive protein was also detected in our case.

In children with COVID-19 infection, radiological findings from chest radiography are usually unspecific (14). Multilobe involvement and a peripheral distribution of lung lesions were noted along with consolidation with a surrounding halo. Ground-glass opacities, more obvious in the chest CT, were seen in one-third of children (9, 14).

Regarding the histological picture of the lung, it corresponded to the few reported cases of COVID-19 infection for which positivity was proven through real-time PCR assay (6, 15). Similar to our case, in autopsy cases from adult COVID-19 patients, the lung parenchyma are described as severely damaged with mononuclear cell infiltration of the alveolar septa, desquamation of pneumocytes, and the genesis of hyaline membrane in the context of acute respiratory distress syndrome (ARDS) (6, 15).

The CD68-positive, giant, multinucleated cells seen in our samples were denominated in the COVID-19-positive cases as “multinucleated syncytial cells” (15). The elongated pneumocytes were considered “viral cytopathic-like changes” (15).

Although the clinically unfavorable evolution of COVID-19 patients was believed to be related to ARDS, the evolution was reported to be even worse than in patients with “classic” ARDS. The present case might offer a new explanation for a poor response to oxygen therapy in similar cases. It is already known that following injury, alveolar denudation of the basal membrane might induce desquamation of CK-positive alveolar epithelial cells/pneumocytes (16). However, the stepwise evolution after desquamation is poorly understood.

It is believed that the desquamated pneumocytes are actively involved in the restoration of alveolar septa or even induce fibrosis via epithelial-mesenchymal transition (16). Although one of our previous studies in patients with ARDS has shown that the hyaline membranes might simultaneously express CD68 (17), the genesis of these membranes was not elucidated. In these cases, the membranes were well-structured, and we did not examine their early stages of genesis. For this reason, we considered them to be formed by hyaline and to induce impairment to oxygen therapy. In the present case, the paradigm consisted of the unusual genesis of the membranes. Based on histological examinations of multi-sliced pulmonary sections and the CD68/CK positivity of the desquamated cells and membranes, we proved that the pneumocytes might become necrotic and induce the encasement of the alveolar septa (Figure 3). In a further step, they are likely transformed in hyaline. If this were to be proven in the other cases or in experimental studies, it may guide the therapeutic management of SARS-CoV-like DIP down a new pathway.

The T-cell-rich red pulp of the spleen and the T-cell hyperplasia of the mesenteric lymph nodes proved, like COVID-19, the hypothesis of T lymphocyte-mediated immune response in this case (15).

Regarding the other organs, in COVID-19-positive adults, autopsy examination has revealed mild hepatic fatty change and inflammatory infiltrate inside the portal spaces (15). In our case, hepatomegaly was associated with hypoxia-induced centrilobular fatty change, which corresponds to the previously reported morphological changes. SARS-CoV-2 infection might also induce non-significant morphological changes in the myocardium such mild mononuclear cell infiltrates (15), but this is not a fact (6, 15). In children, fatal evolution was described to be induced by abnormal coagulation, encephalopathy, and heart and acute kidney failure, which were confirmed at autopsy (7, 8). In our case, kidney failure was induced by the IgA glomerulonephritis, and passive hyperemia could be the result of blood flow impairment through a possible thrombus.

The low incidence of COVID-19 in children is explained by the immaturity of the immune system along with the simultaneous presence of other viruses in the upper and lower airway mucosa that could hinder the growth of SARS-CoV-2 (18–20). In the case presented in this paper, influenza and RSV infection were also excluded. Due to the differences in angiotensin-converting enzyme expression or the paucity of symptoms, glomerulonephritis in this age group can remain undiagnosed and be clinically confused with signs of malnutrition (18–20).

This case highlights the difficulties of diagnosis that occur in the evaluation of children with respiratory disorders, particularly in the COVID-19 era. It also highlights the importance of autopsy in such cases.

### Study Limitations

A limitation of this case is that we did not have serological testing for COVID-19, as it is recognized that even if PCR is negative, evidence of seroconversion can suggest infection.

## Data Availability Statement

The raw data supporting the conclusions of this article will be made available by the authors, without undue reservation.

## Ethics Statement

Ethical review and approval was not required for the study on human participants in accordance with the local legislation and institutional requirements. Written informed consent to participate in this study was provided by the participants' legal guardian/next of kin.

## Author Contributions

SG drafted the paper and performed the autopsy. CS contributed to grossing and histopathological assessment. LM performed the clinical interpretation of the case and contributed to literature review. AS-C and DO contributed to molecular assessment and literature review. BC performed the clinical management. CP and JS conferred the staff support for performing examinations and supervised the management of the case. IJ contributed to histological and immunohistochemical assessment and conferred the final agreement for publication. All authors contributed to the article and approved the submitted version.

## Conflict of Interest

The authors declare that the research was conducted in the absence of any commercial or financial relationships that could be construed as a potential conflict of interest.
